# Polymicrobial Nature of Chronic Diabetic Foot Ulcer Biofilm Infections Determined Using Bacterial Tag Encoded FLX Amplicon Pyrosequencing (bTEFAP)

**DOI:** 10.1371/journal.pone.0003326

**Published:** 2008-10-03

**Authors:** Scot E. Dowd, Randall D. Wolcott, Yan Sun, Trevor McKeehan, Ethan Smith, Daniel Rhoads

**Affiliations:** 1 Research and Testing Laboratory, Lubbock, Texas, United States of America; 2 Southwest Regional Wound Care Center, Lubbock, Texas, United States of America; 3 Medical Biofilm Research Institute, Lubbock, Texas, United States of America; Tufts University, United States of America

## Abstract

**Background:**

Diabetic extremity ulcers are associated with chronic infections. Such ulcer infections are too often followed by amputation because there is little or no understanding of the ecology of such infections or how to control or eliminate this type of chronic infection. A primary impediment to the healing of chronic wounds is biofilm phenotype infections. Diabetic foot ulcers are the most common, disabling, and costly complications of diabetes. Here we seek to derive a better understanding of the polymicrobial nature of chronic diabetic extremity ulcer infections.

**Methods and Findings:**

Using a new bacterial tag encoded FLX amplicon pyrosequencing (bTEFAP) approach we have evaluated the bacterial diversity of 40 chronic diabetic foot ulcers from different patients. The most prevalent bacterial genus associated with diabetic chronic wounds was *Corynebacterium* spp. Findings also show that obligate anaerobes including *Bacteroides*, *Peptoniphilus*, *Fingoldia*, *Anaerococcus*, and *Peptostreptococcus* spp. are ubiquitous in diabetic ulcers, comprising a significant portion of the wound biofilm communities. Other major components of the bacterial communities included commonly cultured genera such as *Streptococcus*, *Serratia*, *Staphylococcus* and *Enterococcus* spp.

**Conclusions:**

In this article, we highlight the patterns of population diversity observed in the samples and introduce preliminary evidence to support the concept of functional equivalent pathogroups (FEP). Here we introduce FEP as consortia of genotypically distinct bacteria that symbiotically produce a pathogenic community. According to this hypothesis, individual members of these communities when they occur alone may not cause disease but when they coaggregate or consort together into a FEP the synergistic effect provides the functional equivalence of well-known pathogens, such as *Staphylococcus aureus*, giving the biofilm community the factors necessary to maintain chronic biofilm infections. Further work is definitely warranted and needed in order to prove whether the FEPs concept is a viable hypothesis. The findings here also suggest that traditional culturing methods may be extremely biased as a diagnostic tool as they select for easily cultured organisms such as *Staphylococcus aureus* and against difficult to culture bacteria such as anaerobes. While PCR methods also have bias, further work is now needed in comparing traditional culture results to high-resolution molecular diagnostic methods such as bTEFAP.

## Introduction

Chronic human infections, including chronic wounds, constitute 60–80% of all human infectious diseases [1]. The costs of chronic infections represent a major portion of the healthcare budget and these costs continue to grow at exponential rates [2].

Diabetic extremity ulcers develop in approximately 15 percent of people with diabetes and are a leading cause of hospitalization and amputation among such patients [3]. Wound infection, faulty wound healing, and ischemia in combination with a foot ulcer are the most common precursors to diabetes-related amputations; and eighty-five percent of lower-limb amputations in patients with diabetes are preceded by biofilm infected foot ulceration [4]–[6]. More than 80,000 amputations are performed on the United States' diabetic population each year [7]. Diabetic foot ulcer infection followed by amputation contribute dramatically not only to the morbidity among persons with diabetes [8] but are also associated with severe clinical depression and dramatically increased mortality rates [9]. Such infected ulcers resulting in amputation account for a threefold increased risk of death within 18 months. Additionally, the psychological impact of an amputation dramatically increases this risk of mortality within a similar time period. As such, diabetic foot ulcers are the most common, disabling, and costly complications of diabetes [10], [11].

A primary impediment to the healing of chronic wounds is biofilm phenotype infections [12]–[14]. Biofilms, by definition, are the ubiquitous and natural phenotype of bacteria. They typically consist of polymicrobial populations of cells, which are attached to a surface and encase themselves in hydrated extracellular polymeric substances. “Microbial populations that have attached to a biological or non-biological surface” is the most basic description of a medical biofilm. Thus, most chronic infections, including bacterial that are associated with chronic wounds, exist as biofilm communities [13], [15]–[17]. Bacteria that reside within mature biofilms are highly resistant to many traditional therapies. Currently, one of the most successful strategies for the management of biofilm-related conditions is physical removal of the biofilm, such as frequent debridement of the diabetic foot ulcers [12], [13], [18], [19].

A notable wound care committee [3] has recently suggested that microbiological investigations of wound infections are of limited use for diagnosing most infections. The limited advantages from culturing are due to the length of time required to obtain results. Traditional culturing takes days to obtain results, but molecular microbial studies can often be performed within 24 hours. The primary care suggestion for wounds is to obtain results from microbial analysis before commencing empiric antibiotic therapy. Because of this only molecular methods have the ability to identify the bacteria within chronic wounds in a timeframe conducive to true medical paradigms of evaluate, diagnose, treat, reevaluate.

Little is known about the types of bacteria that might contribute to the bioburden in diabetic foot ulcers. There have been only a few recent surveys of bacterial populations associated with various chronic wounds as reviewed and investigated by our group [13], [17]. Traditionally, microbial studies of the wound microbiota have focused on the role of easily cultured and well-known pathogens such as *Staphylococcus aureus* and *Pseudomonas aeruginosa*. These organisms are cultured easily using traditional microbiological evaluations, and, therefore, standard methods likely overestimate the contribution of these species to the microbiota of chronic wounds. Now, the medical and research communities are beginning to realize that the diversity of bacterial populations in chronic wounds may be an important contributor to the chronicity of wounds, such as diabetic foot ulcers. The current study was undertaken in an attempt to examine the major populations of bacteria associated with the bioburden of infected diabetic foot ulcers. By performing a survey of wounds from different subjects, we hoped to identify genera or noted pathogens that are consistently present in diabetic ulcers. Alternatively, we may identify functionally equivalent symbiotic consortia of bacteria that are associated with chronic wounds.

## Methods

### Diabetic wound samples

Debridement samples were collected from 40 subjects at the Southwest Regional Wound Care Center (Lubbock, Texas) in accordance with Western Institutional Review Board protocol number 20062347. All patients provided written consent. Subjects were chosen were chosen for this study who had diabetic extremity ulcers on their feet. The wounds were from lateral (n = 8), dorsal (n = 1), plantar (n = 8), ankle (n = 2), 5th metatarsal head (n = 3), 4th metatarsal head (n = 2), 2nd Metatarsal head (n = 2), trans metatarsal (n = 1), 1st metatarsal head (n = 3), Great toe (n = 3), heel (n = 7). Sharp debridement samples were collected with sterile tools using sterile technique and immediately frozen in collection tubes at −80°C until DNA extraction was performed as described previously [17].

### DNA extraction

After thawing, portions of the debridement (200 mg±100 mg) were recovered using sterile forceps. The samples were placed in 2 ml sterile micro centrifuge tubes. Samples were centrifuged at 14,000 rpm for 30 seconds and resuspended in 500 µl RLT buffer (QIAGEN, CA, USA) (with β- mercaptoethanol). A sterile 5 mm steel bead (QIAGEN, CA, USA) and 500 µl 0.1 mm glass beads (Scientific Industries, Inc., NY, USA) were added for complete bacterial lyses in a Qiagen TissueLyser (QIAGEN, CA, USA), run at 30 Hz for 5 min. Samples were centrifuged briefly, and 100 µl 100% ethanol were added to a 100 µl aliquot of the sample supernatant. This mixture was added to a DNA spin column, and DNA recovery protocols were followed as instructed in the QIAamp DNA Mini Kit (QIAGEN, CA, USA) starting at step 5 of the Tissue Protocol. DNA was eluted from the column with 30 µl water and samples were diluted accordingly to a final concentration of 20 ng/µl for use with SYBR Green RT-PCR (Qiagen, Valencia, CA). DNA samples were quantified using a Nanodrop spectrophotometer (Nyxor Biotech, Paris, France).

### PCR to create tag encoded amplicons

All DNA samples were diluted to 100 ng/µl. A 100 ng (1 µl) aliquot of each sample's DNA was used for a 50 µl step 1 PCR reaction. The 16S universal Eubacterial primers 530F (5′-GTG CCA GCM GCN GCG G) and 1100R (5′-GGG TTN CGN TCG TTG) were used for amplifying the 600 bp region of 16S rRNA genes. HotStarTaq Plus Master Mix Kit (Qiagen, Valencia, CA) was used for PCR under the following conditions: 94°C for 3 minutes followed by 30 cycles of 94°C for 30 seconds; 60°C for 40 seconds and 72°C for 1 minute; and a final elongation step at 72°C for 5 minutes. A step 2 PCR was performed for 454 amplicon sequencing under the same condition by using designed special fusion primers with different tag sequences as described previously [20]. The use of a secondary PCR prevents amplification of some biases caused by inclusion of tag and linkers during initial template amplification reactions. After secondary PCR, all amplicon products from different samples were mixed in equal volumes, and purified using Agencourt Ampure beads (Agencourt Bioscience Corporation, MA, USA).

### Massively parallel bTEFAP using a FLX

In preparation for FLX sequencing (Roche, Nutley, New Jersey), the DNA fragments' size and concentration were accurately measured by using DNA chips under a Bio-Rad Experion Automated Electrophoresis Station (Bio-Rad Laboratories, CA, USA) and a TBS-380 Fluorometer (Turner Biosystems, CA, USA). A 9.6 E+06 sample of double-stranded DNA molecules/µl with an average size of 625 bp were combined with 9.6 million DNA capture beads, and then amplified by emulsion PCR. After bead recovery and bead enrichment, the bead-attached DNAs were denatured with NaOH, and sequencing primers were annealed. A two-region 454 sequencing run was performed on a 70×75 GS PicoTiterPlate (PTP) using the Genome Sequencer FLX System (Roche, Nutley, New Jersey). Twenty tags were used on region of the PTP. All FLX procedures were performed using Genome Sequencer FLX System manufacturer's instructions (Roche, Nutley, New Jersey).

### bTEFAP sequence processing pipeline

Custom software written in C# within a Microsoft®.NET (Microsoft Corp, Seattle, WA) development environment was used for all post sequencing processing. Discussion of software code is outside the scope of this report; however, a brief description of the algorithm follows. Quality trimmed sequences obtained from the FLX sequencing run were derived directly from FLX sequencing run output files. Tags were extracted from the multi-FASTA file into individual sample-specific files based upon the tag sequence. Tags which did not have 100% homology to the sample designation were not considered. Sequences which were less than 150 bp after quality trimming were not considered. The resultant individual samples were assembled using CAP3 after parsing the tags into individual FASTA files [21]. The ace files generated by CAP3 were then processed to generate a secondary FASTA file containing the tentative consensus (TC) sequences of the assembly along with the number of reads integrated into each consensus. TC were required to have at least 2-fold coverage. The resulting TC FASTA for each sample was then evaluated using BLASTn [22] against a custom database derived from the RDP-II database [23] and GenBank (http://ncbi.nlm.nih.gov). The sequences contained within the curated 16S database were both >1200 bp and considered of high quality based upon RDP-II standards. A post processing algorithm generated best-hit files with E-values <10e-114 and bit scores >400. The identities of all hits were greater than 98%. These parameters, based upon an average TC length of 260 bp, have been previously evaluated to enable reliable identification at the genus level [17]. Identification at the species level should only be considered putative and for the purpose of this pilot study, species designations have been ignored. Following best-hit processing a secondary post-processing algorithm was used to combine genus designations generating a list of genera IDs and their relative predicted abundance within the given sample.

### Statistics

Basic statistics were performed using the Basic comparative functions and multivariate hierarchical clustering methods of JMP 6.0 (SAS institute, Cary, NC).

## Results and Discussion

### Bacterial diversity in wounds

Our group was the first to use bTEFAP-like pyrosequencing approach in the evaluation of chronic wound microbiota [17]. In our previous study, we conducted a broad survey of wounds using a variety of molecular methods and concluded that the bacterial communities in diabetic foot ulcers had a high degree of diversity [17]. The most prevalent populations of bacteria identified in the previous study, which surveyed a single pooled sample from 10 diabetic patients, in order, were *Staphylococcus*, *Peptoniphilus*, *Pseudomonas*, *Anaerococcus*, *Enterococcus*, *Bacteroides*, *Veillonella*, *Finegoldia*, and *Clostridium* spp.

The current study uses the same broad survey approach as the previous study. However, instead of a single pool of bacteria from multiple diabetic foot ulcers, the current study evaluates the bacteria in 40 individual diabetic foot ulcers. We hypothesized that a single major pathogen such as *Staphylococcus aureus* would be associated with all such wounds, while our alternative hypothesis was that there would be no single pathogen associated with these samples. The latter hypothesis would suggest that a mixed-species biofilm (not a lone pathogen) causes the chronic infection observed in longstanding diabetic foot ulcers. This new concept of functionally equivalent pathogroup (FEP) populations suggests individual traditionally “non-pathogenic” species may live symbiotically and act synergistically, thereby contributing to or causing the chronicity of diabetic foot wounds.

The current study disproved the original hypothesis that a lone species is the common culprit in these chronic infections. No single genus of bacteria was present in all of the diabetic foot ulcers. The most ubiquitous genus was *Corynebacterium*, which was found in 30 of the 40 diabetic ulcers. *Bacteroides*, *Peptoniphilus*, *Fingoldia* and *Anaerococcus* spp. were also highly prevalent and were present in 25, 25, 23, and 22 of the samples respectively. Table 1 details all of the genera that occurred in at least 5% (2 of 40) of the diabetic ulcers. The table also includes the number of samples that contained each genus, the average contribution of each genus to the total bacterial population in those samples as represented as a percentage, the corresponding range of percentages, and the corresponding standard deviations. In our previous survey [17], the primary organism detected was *Staphylococcus*. Interestingly, this genus was only detected in 13 of the 40 samples in the current study. These apparently dissimilar results are likely an effect of pooling the samples in the previous study where one or more of the samples pooled may have had an abundance of *Staphylococcus*, which would have decreased the representation of the other genera in the pool. In the current study, each of the samples was individually analyzed using bTEFAP [20], which enabled us to remove the bias of pooling samples.

**Table 1:** Bacterial genera identified in 40 diabetic foot ulcers.

Genera	Samples	Avg %	Std dev	Min-max %
*Corynebacterium* spp.	30	14.4	27.5	0.22–80.6
*Bacteroides* spp.	25	24.2	34.8	0.15–98.8
*Peptoniphilus* spp.	25	13.6	9.9	0.22–38.4
*Finegoldia* spp.	23	6.7	4.1	0.65–20.5
*Anaerococcus* spp.	22	7.7	6.1	1.28–23.8
*Streptococcus* spp.	21	36.5	26.2	1.68–88.8
*Serratia* spp.	17	21.4	22.9	0.82–98.4
*Unknown-b*	15	16.8	13.2	0.93–62.2
*Staphylococcus* spp.	13	8.3	10.0	0.65–32.6
*Prevotella* spp.	12	7.4	24.9	0.87–37.3
*Peptostreptococcus* spp.	11	8.7	4.5	0.85–41.5
*Porphyromonas* spp.	10	7.0	3.6	2.38–24.3
*Enterococcus* spp.	10	2.8	1.2	0.31–8.4
*Actinomyces* spp.	9	5.7	5.6	1.81–20.2
*Pseudomonas* spp.	8	14.5	11.6	0.67–94.3
*Clostridium* spp.	8	2.3	3.2	0.75–5.9
*Helcococcus* spp.	5	2.5	3.0	0.91–7.3
*Brevibacterium* spp.	5	1.8	0.7	0.71–2.46
*Varibaculum* spp.	4	9.0	10.5	1.46–27.8
*Aerococcus* spp.	4	3.0	3.6	0.47–7.0
*Fusobacterium* spp.	3	5.6	2.6	1.99–7.9
*Arthrobacter* spp.	3	3.8	2.5	1.85–7.4
*Bacillus* spp.	3	3.5	3.0	0.19–7.5
*Anaerobiospirillum* spp.	3	2.3	1.1	0.37–3.8
*Actinobaculum* spp.	3	1.9	1.0	0.53–2.9
*Dermabacter* spp.	3	1.6	0.9	0.78–2.87
*Salmonella* spp.	3	1.5	1.1	0.52–3.02
*Veillonella* spp.	3	1.3	4.5	1.12–1.49
*Citrobacter* spp.	2	9.5	2.5	7.0–12.0
*Rothia* spp.	2	5.8	3.2	1.27–10.2
*Tissierella* spp.	2	4.0	2.7	1.34–6.6
*Propionibacterium* spp.	2	3.3	0.4	2.82–3.7
*Proteus* spp.	2	3.1	2.2	0.89–5.3
*Aerosphaera* spp.	2	2.8	1.9	1.11–4.5
*Peptococcus* spp.	2	2.5	0.8	1.64–3.3
*Dermabacter* spp.	2	1.2	0.7	0.61–1.85
*Granulicatella* spp.	2	1.2	0.3	0.86–1.51
*Brevundimonas* spp.	2	0.9	0.2	0.63–1.07

The genera identified in the current study of bacterial populations in 40 different diabetic foot ulcers are reported. The genera are sorted by the number of samples in which they were detected (Samples). The average percentage each genus contributed to its positive samples is noted (Avg %), as well as the standard deviation of the percentages (Std dev) and the range of percentages (Min-max %).

*Corynebacterium* was the predominant genus identified in this study. The tentative identification of the species includes *C. striatum*, *C. amycolatum*, *C. tuberculostearicum*, and *C. mucifaciens* in order of occurrence. *Corynebacterium striatum* was identified in 22 of the 30 *Corynebacterium*-positive samples. This species has been associated with infections involving joints and open fracture wounds [24]. Another study has associated *Corynebacterium* with diabetic foot osteomyelitis [25]. In the noted study, the evaluation was performed using traditional culturing methods, which identified the fastidious *Corynebacterium*, which is commonly considered a contaminant. In contrast, the easily cultured *Staphylococcus aureus* is commonly considered the primary pathogen in these samples. It must be indicated that there are inherent bias associated with molecular methods such as bTEFAP as discussed in our previous work [20]. Yet we suggest that culture techniques have a much more dramatic bias and greatly overestimate the importance of organisms that are easily cultured and can underestimate the importance of fastidious organisms, which require more specialized culture methods [26].

In another study, Wheat *et al* [27] use an advanced approach to culturing bacteria, and their results support our findings. They discovered *Staphylococcus*, *Enterococcus*, and *Corynebacterium* were the most common aerobic (or facultative) bacteria in foot ulcers. They also did an excellent job of evaluating anaerobes in foot ulcers and identified common anaerobes such as *Peptostreptococcus* and *Bacteroides* species, which also agrees with our results (Table 1). Other studies [28], [29] have identified the importance of *Corynebacterium* in foot ulcers. This genus may be considered to be nonpathogenic because it is “normal flora” of human skin and mucous membranes, but bacteria such as *Staphylococcus epidermidis* had been previously been mistakenly viewed as nonpathogenic for the same reason [30]. Although *Corynebacterium* may not be a common cause of acute infections, it appears to be a common (but overlooked) player in chronic diabetic foot ulcer infections [31]. We suggest that a shift in the methods of the clinical microbiology laboratory would help to overcome this bias. It is appropriate to routinely use molecular techniques such as PCR or bTEFAP [20] that are more reliable than traditional culturing methods. By using approaches that do not rely on the ability of bacteria to grow well in culture, we can begin to have a less biased view of the bacterial diversity present in chronic wounds, and we can reexamine the clinical prejudices associated with the presence of “nonpathogenic” bacteria.

The occurrence of anaerobes in wounds has also been well documented in the literature, and it suggests that anaerobes are beginning to be recognized as major populations in chronic wound biofilms [17], [32]–[36]. The importance of anaerobes such as *Peptostreptococcus*, *Prevotella*, *Finegoldia* and *Peptoniphilus* have been previously reported [32], [37]–[41], and the present study agrees that these genera represent a significant portion of diabetic ulcer microbiota. Even though wounds are typically exposed to air [32], anaerobes may be the most prevalent physiological type for a given wound or an individual wound type. Bowler *et al* [32] evaluated venous leg ulcers using cultural isolation techniques that included special considerations for the propagation of anaerobes. They reported that anaerobes represented 49% of the total microbial composition in such wounds. Dowd *et al* [17], using a pyrosequencing approach, reported that 30% of the sequences from pooled diabetic ulcers were anaerobes.

### Functionally equivalent pathogroups (FEPs)

The comorbidities associated with the pathophysiology of a given wound may create a definable host condition. The host condition and the host's environment may work together to influence the ecology of the wound. Patients with similar comorbidities and similar environmental conditions may share common wound ecology. Each ecological wound type may be defined by the unique bacterial consortium, which it supports. If this is true, research should be performed to link common bacterial consortia with the common wound ecosystem and the unique clinical management strategies capable of collapsing each type of system. We propose that the wound's microbiota is a key component that delays healing, and collapsing the microbial ecosystem would thereby expedite the healing process.

**Figure 1:**
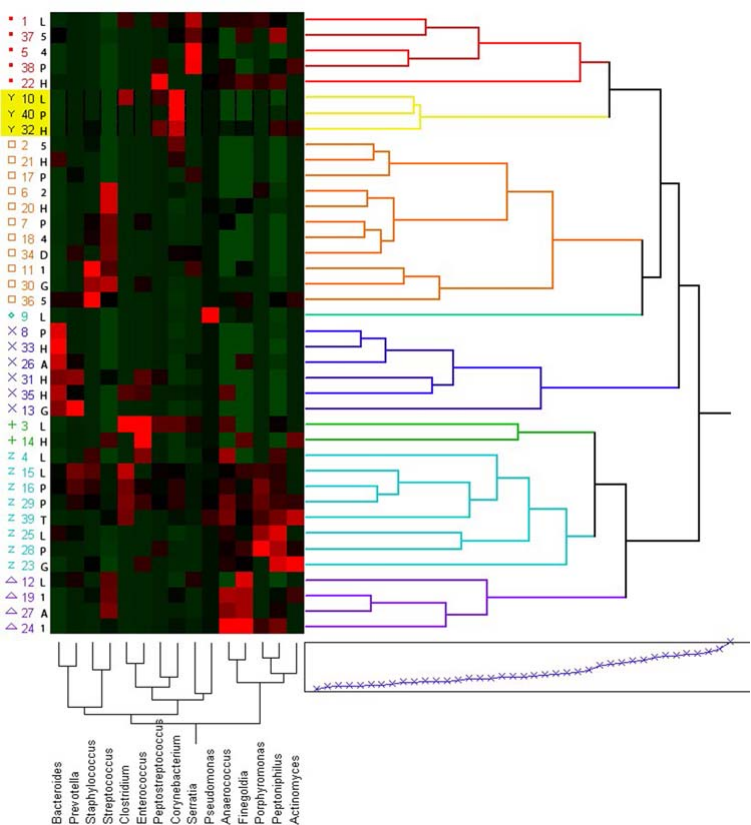
Dendrogram of Functional Equivalent Pathogroups (FEPs). The most prevalent bacterial genera were used to perform multivariate hierarchical clustering. Using a geographic X scale and a color map we represent the 40 different wounds along the Y-axis and the predominant genera along the X-axis in this dendogram, which shows 8 primary clusters associated with possible functional equivalent pathogroups (FEPs). Thus, in cluster 1 (red dots) we see that the predominant genera are *Serratia* spp. and anaerobes (*Finegoldia*, *Peptoniphilus* and *Anaerococus* spp.). Together these genera contribute to FEP in cluster 1. Cluster 2 (yellow Y's) is made up of *Corynebacterium* and the same anaerobes as cluster 1. The most predominant cluster, cluster 3 (orange squares), involves *Streptococcus* and anaerobes including the previously mentioned genera from clusters 1 and 2 as well as *Bacteroides*. Cluster 4 (green diamond) is only a single sample but involves co-occurrence of *Pseudomonas*, *Streptococcus* and *Porphyomonas* spp. Cluster 5 (blue x's) is heavily populated by anaerobes particularly *Bacteroides*, *Anaerococcus*, *Fingoldia*, and *Peptoniphilus* spp. Cluster 6 (green crosses) is only made up of two samples and includes *Enterococcus* as the primary organism with significant signatures from *Anaerococcus*, *Finegoldia*, and *Peptoniphilus* spp. Cluster 7 (blue Z's) has the strongest color map signatures associated with anaerobes, especially *Clostridium*, *Fingoldia*, *Porphyromonas* and *Peptoniphilus* spp. Finally, cluster 8 is strongly associated with the anaerobes *Anaerococcus* and *Fingoldia* spp. with additional contributions from *Streptococcus* spp. The location of each extremity ulcer is also encoded into this figure along the Y-axis. The codes for the wound locations are lateral foot ulcer (L), dorsal foot ulcer (D), plantar foot ulcer (P), ankle ulcer (A), 5th metatarsal head ulcer (A), 4th metatarsal head ulcer (4), 2nd Metatarsal head ulcer (2), trans metatarsal ulcer (T), 1st metatarsal head ulcer (1), Great toe ulcer (G), and heel ulcer (H).

To begin to evaluate the co-occurrence of particular species or to identify if there are multi-species microbial patterns associated with chronic wound infections, we used multivariate hierarchical clustering functions for those bacteria genera that were detected in greater than 10% of the wounds. This resulted in 8 major clusters that we term functional equivalent pathogroups (FEP). Figure 1 shows a two-dimensional dendogram depicting these clusters. Our observations yielded a hypothesis that has yet to be formally tested. We hypothesize that certain bacterial species may not be capable of maintaining a chronic infections on their own, but if these species co-occur in appropriate mixtures, they can act symbiotically to successfully establish a pathogenic biofilm, which contributes to the chronicity of the wound.

Using the data represented in Figure 1, we can identify prominent anaerobic FEP including cluster 5, cluster 7, and cluster 8. These clusters may be some of the more important FEP associated with diabetic ulcers, but they are not easily identified using traditional culturing methods. Anaerobes also appear to be a common thread that links all FEP, and this study highlights the importance of performing further studies that examine the role that anaerobes may play in promoting the chronicity of diabetic foot ulcers and other wounds.

As discussed previously [42], it has been demonstrated in the laboratory [43], [44] that obligate anaerobes may cope with the toxic effects of oxygen by interacting with aerobic or facultative bacteria populations in a symbiotic manner as part of a process known as coaggregation. Aerobic species may consume oxygen and create localized niches, allowing the obligate anaerobes to gain an advantage when in close proximity to their oxygen-reducing neighbors. The Lewandowski lab has also shown that oxygen only penetrates microns into the surface of biofilms, which suggests that internal regions of the bacterial communities may support only anaerobes and facultative anaerobes [45]. The symbiotic interactions between different bacterial species and the advantages associated with the biofilm phenotype have been demonstrated for specific aerobes and anaerobes.

Specific mixtures of anaerobic and aerobic bacteria in animal models produce disease states which cannot be reproduced by individual species [34], [35], [46]–[48], [48], [49]. Similar to previous work showing gut and oral consortial etiologies, our findings provide a added perspective to Koch's postulates and suggest a complexity of host-pathogen interaction that traditional culturing does not reveal. Our findings highlight the shortcoming of relying on culture methods to identify the important bacterial populations within clinical samples, and our results suggest that identifying and understanding the bacterial FEPs in chronic wounds may be needed in order to better manage these infections.

It has been proposed that sequencing the 16S gene of clinical, laboratory-cultivated bacteria is advantageous over the traditional biochemical identification methods [50]. We propose that not only is a molecular sequencing approach better for the identification of cultivated microbes, but also sequencing can be used to identify organisms without the need to culture. The culturability of pathogens has been at the central dogma of medical microbiology since its inception, but in this molecular age, it is now possible and necessary to step beyond this traditional methodology in an attempt to unravel the complexity of chronic, mixed infections. Using bTEFAP, it is now possible (and will soon be practical) to avoid the biased culture methods currently used to identify the bacteria in clinical samples.

Functional equivalence, in relation to biofilm and the bacterial populations found in chronic wounds, is an important concept that may dramatically alter the single-pathogen paradigm of chronic infections. For instance, we may discover that *Corynebacterium* spp. and anaerobes such as *Fingoldia* and *Peptoniphilus* spp. work together to create a pathogenic group (FEP) that is equivalent to commonly noted pathogens such as *Pseudomonas aeruginosa* or *Staphylococcus aureus*. Diverse pathogenic biofilms are more stable than less diverse biofilms [51], and the presence of a single, predominant pathogenic species in a chronic infection is arguably naive [52]. Our current and previous work [13], [17] indicates the strong possibility and validity of the “functional equivalence” hypothesis. The presence of FEPs could explain the recalcitrance of some wounds when managed using a single treatment, and a high diversity of bacterial species may explain why multiple concurrent strategies benefit the course of chronic wound healing [14].

For diverse species to work as a whole to collectively produce a persistent infection, they must possess the properties that allow single pathogens to be successful. Thus, these diverse consortia must possess “functional equivalence” which allows the whole to establish and maintain the chronic infection. Whereas, pathogens such as *P. aeruginosa* may be capable of performing all of the essential roles required for pathogenesis, FEPs may distribute these roles among multiple species. In a single FEP, at least one representative species within the consortia must be able to perform each essential role. The significance of this hypothesis may prove important in developing improved methods for diagnosing and treating chronic wound infections.
